# Supplementary figures and images for: A high quality assembly of the Nile Tilapia (Oreochromis niloticus) genome reveals the structure of two sex determination regions
Source: BMC Genomics. 2017 May 2;18:341. doi: 10.1186/s12864-017-3723-5 (PMC5414186; doi:10.1186/s12864-017-3723-5)

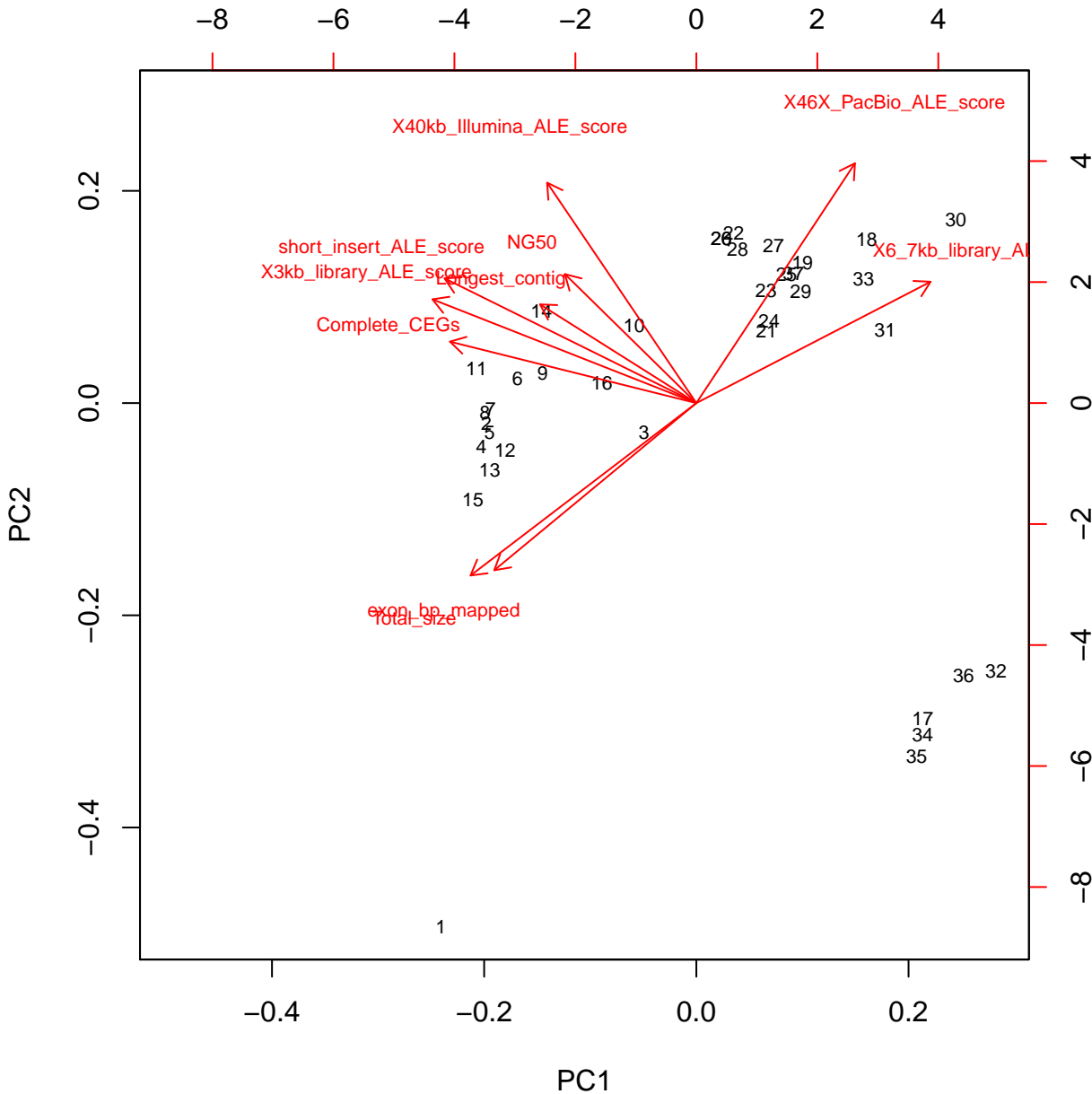

Supplement: Supplementary file 2 — PCA analysis of candidate assemblies. PCA analysis of the 37 candidate assemblies (assembly numbers listed in Additional file 1 and Additional file 5) is composed of total size (bp), exon bp mapped, complete CEGMA CEGs, NG50, longest contig, and overall ALE scores (Illumina fragment, 3kbp, 6–7kbp, 40kbp and 44X PacBio libraries). (PDF 5 kb) [file 12864_2017_3723_MOESM2_ESM.pdf]

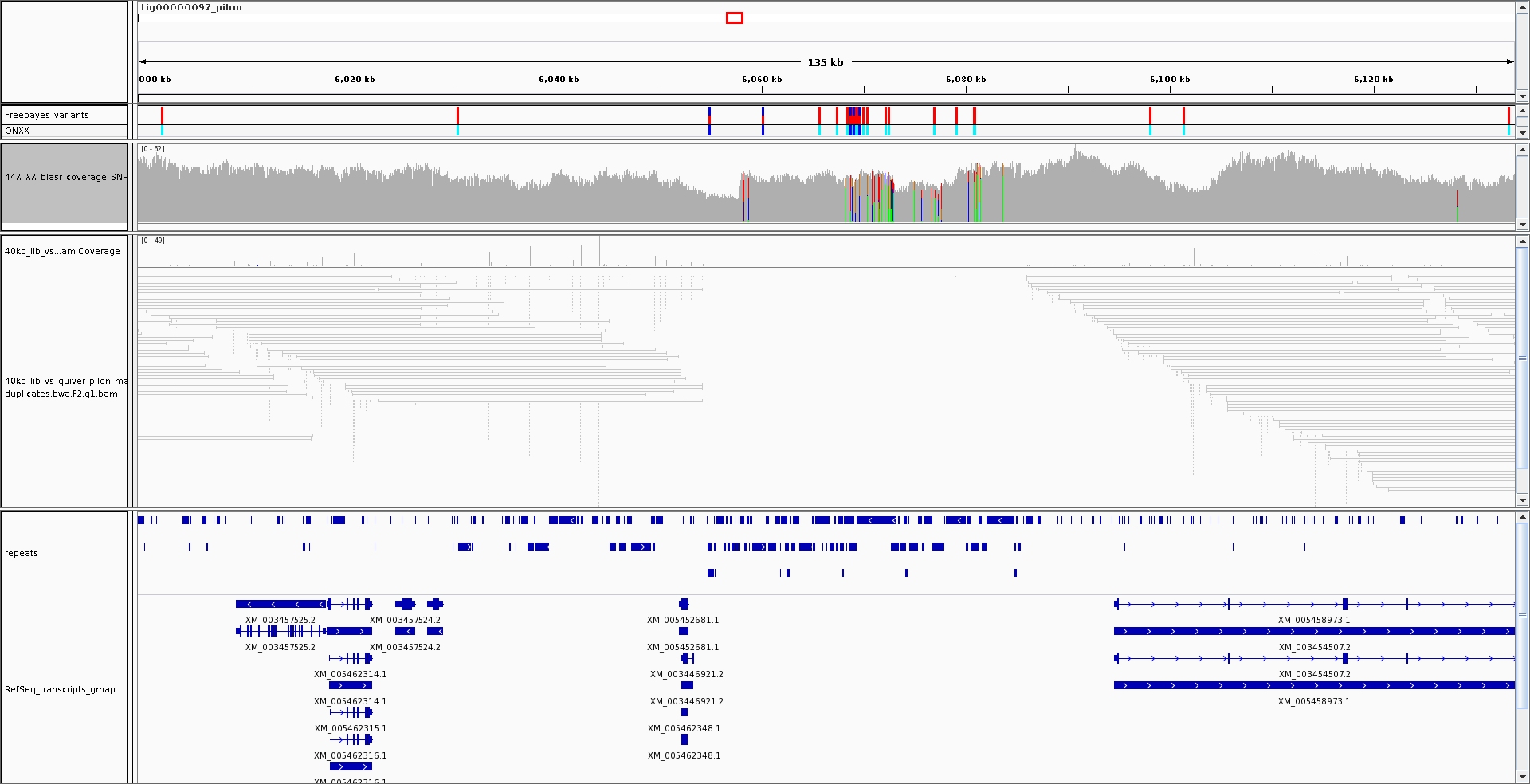

Supplement: Supplementary file 3 — Example misassembly signature. An example misassembly identified by both RH and RAD maps showing the characteristic signature of high variation in the 44X PacBio read alignments as well as low coverage in the 40kbp Illumina mate-pair library and high density of repetitive elements. (PNG 37 kb) [file 12864_2017_3723_MOESM3_ESM.png]
